# Association of immuno-inflammatory biomarkers with response to neoadjuvant chemotherapy and prognosis in HER2-positive breast cancer: dual-center clinical evidence

**DOI:** 10.3389/fimmu.2026.1751072

**Published:** 2026-02-09

**Authors:** Xuanlin Wu, Siqi Wu, Mankai Tang, Longgui Xie, Jianhui Liu, Jiexu Liao, Jie Wang, Huawei Yang

**Affiliations:** Department of Breast Surgery, Key Laboratory of Breast Cancer Diagnosis and Treatment Research of Guangxi Department of Education, Guangxi Medical University Cancer Hospital, Nanning, China

**Keywords:** correlation analysis, disease-free survival, neoadjuvant chemotherapy, pathological complete response, systemic immune-inflammation index

## Abstract

**Purpose:**

Peripheral blood immuno-inflammatory biomarkers (IIBs) may help predict response to neoadjuvant chemotherapy (NAC) and prognosis in HER2-positive breast cancer. This study compared the predictive value of neutrophil-to-lymphocyte ratio (NLR), monocyte-to-lymphocyte ratio (MLR), platelet-to-lymphocyte ratio (PLR), and systemic immune-inflammation index (SII) for pathological complete response (pCR) and disease-free survival (DFS).

**Patients and methods:**

A total of 224 female patients with HER2-positive invasive breast cancer who received NAC followed by surgery at two medical centers (2015–2023) were retrospectively analyzed. Baseline IIBs were calculated from complete blood counts. Receiver operating characteristic (ROC) curves identified optimal cut-offs. Logistic and Cox regression analyses combined with the least absolute shrinkage and selection operator (LASSO) method were used to determine factors associated with pCR and DFS. Subgroup analyses were performed to assess consistency across clinical and treatment variables.

**Results:**

SII demonstrated the highest discriminatory ability among tested IIBs for predicting pCR (AUC = 0.739) and was significantly associated with longer DFS (P < 0.001). Patients with low SII had higher pCR rates and improved DFS. These associations remained stable across prespecified subgroups. Other factors related to better response included lower CA15-3/CEA levels, ≥6 NAC cycles, receipt of HER2-targeted therapy, and breast-conserving surgery.

**Conclusions:**

Among common inflammatory indices, SII demonstrated the strongest association with treatment response and prognosis in HER2-positive breast cancer. As an inexpensive, readily available biomarker, it may assist clinical risk stratification. However, given the retrospective design and substantial heterogeneity in treatment regimens, these findings should be interpreted cautiously and validated in prospective studies.

## 1 Introduction

Breast cancer remains the most prevalent malignancy among women worldwide, with persistently high incidence and mortality rates posing a major global health challenge (1). Among them, human epidermal growth factor receptor 2 (HER2) positive breast carcinoma accounts for approximately 15-20% of all cases. This subgroup has drawn significant attention due to its high aggressiveness and poor prognosis (2). However, with the advent of anti-HER2 targeted agents (such as Trastuzumab and Pertuzumab), the treatment landscape for this molecular subtype has undergone a fundamental transformation. NAC combined with targeted therapy has become the standard treatment modality for locally advanced HER2-positive breast cancer (3). NAC not only facilitates tumor downstaging and increases breast-conserving surgery (BCS) rates, but more importantly, achievement of pCR—defined as the absence of invasive carcinoma in both breast and axillary nodes—has been validated as a robust surrogate endpoint for long-term survival outcomes, including disease-free survival (DFS) and overall survival (OS) (4–6). Despite significant progress, a considerable proportion of patients still exhibit poor response to NAC in clinical practice. This heterogeneity in treatment response underscores the urgent need to identify potential beneficiaries before initiating therapy (7).

To achieve precise individualized treatment, there is an urgent clinical need for a reliable, non-invasive, and easily accessible biomarker to predict NAC efficacy and patient prognosis. An ideal biomarker should enable risk stratification of patients before the initiation of treatment, thereby guiding therapeutic decision-making: for patients predicted to have favorable efficacy, de-escalation of therapy may be considered to reduce toxic side effects; whereas for those predicted to have poor efficacy, more intensive Treatment regimen may be explored, or they may be recommended to participate in a clinical trial. However, widely used clinicopathologic biomarkers—such as estrogen receptor (ER) and progesterone receptor (PR) status or the Ki-67 proliferation index—offer only limited predictive power for NAC responsiveness and require invasive tissue biopsy (8). Therefore, the search for new biomarkers that can reflect the interaction between neoplasms and the host has become a hot research topic.

Increasing evidence indicates that the systemic immune–inflammatory state plays a pivotal role in tumor initiation, progression, metastasis, and treatment response (9). The dynamic cross-talk between the tumor microenvironment and the systemic immune system not only influences the biological behavior of neoplasms but also profoundly affects the efficacy of chemotherapy and targeted therapy. Easily detectable immune cells and inflammatory factors in peripheral blood (such as neutrophils, lymphocytes, and thrombocytes) are regarded as an “early-warning radar system” for this state of balance (10, 11). Inflammatory indices constructed from cell counts obtained based on routine haematology tests, such as the neutrophil-to-lymphocyte ratio (NLR) and the platelet-to-lymphocyte ratio (PLR), are inexpensive, reproducible, and have shown prognostic relevance across multiple solid tumors, including breast cancer (12–14). However, the roles of multiple inflammation-based indices in the neoadjuvant chemotherapy setting for HER2-positive breast cancer have not yet been established.

Systemic immune-inflammation index (SII) is a novel comprehensive indicator that integrates neutrophil, platelet, and lymphocyte counts. Its calculation formula is (Platelet count × Neutrophil count/Lymphocyte count) (15). Conceptually, it captures the net imbalance between pro-tumor inflammatory activity (mediated by neutrophils and platelets) and anti-tumor immune surveillance (mediated by lymphocytes). A high SII value indicates a more active pro-inflammatory environment and a weakened immune surveillance capability, which may diminish the anti-tumor effects of chemotherapy and targeted therapy, and promote neoplasm recurrence (16, 17). While SII has shown superior prognostic value in colorectal, hepatocellular, urothelial, and nasopharyngeal carcinomas (18–21), its role in HER2-positive breast cancer, particularly as a predictive biomarker for post-NAC pCR and long-term outcomes (DFS), has not been fully elucidated or validated.

Therefore, the present study was designed to systematically evaluate and compare pre-treatment SII with other peripheral immune–inflammatory biomarkers (NLR, PLR, and monocyte-to-lymphocyte ratio [MLR]) in the prediction of NAC efficacy (pCR) and prognosis (DFS) among HER2-positive breast cancer patients. We hypothesize that, as a more comprehensive indicator, SII can predict clinical outcomes more accurately and stably than other traditional indicators, thereby providing a simple and effective new tool for risk stratification and individualized treatment decision-making in this specific patient population.

## 2 Materials and methods

### 2.1 Cohort and data collection

**Figure 1:**
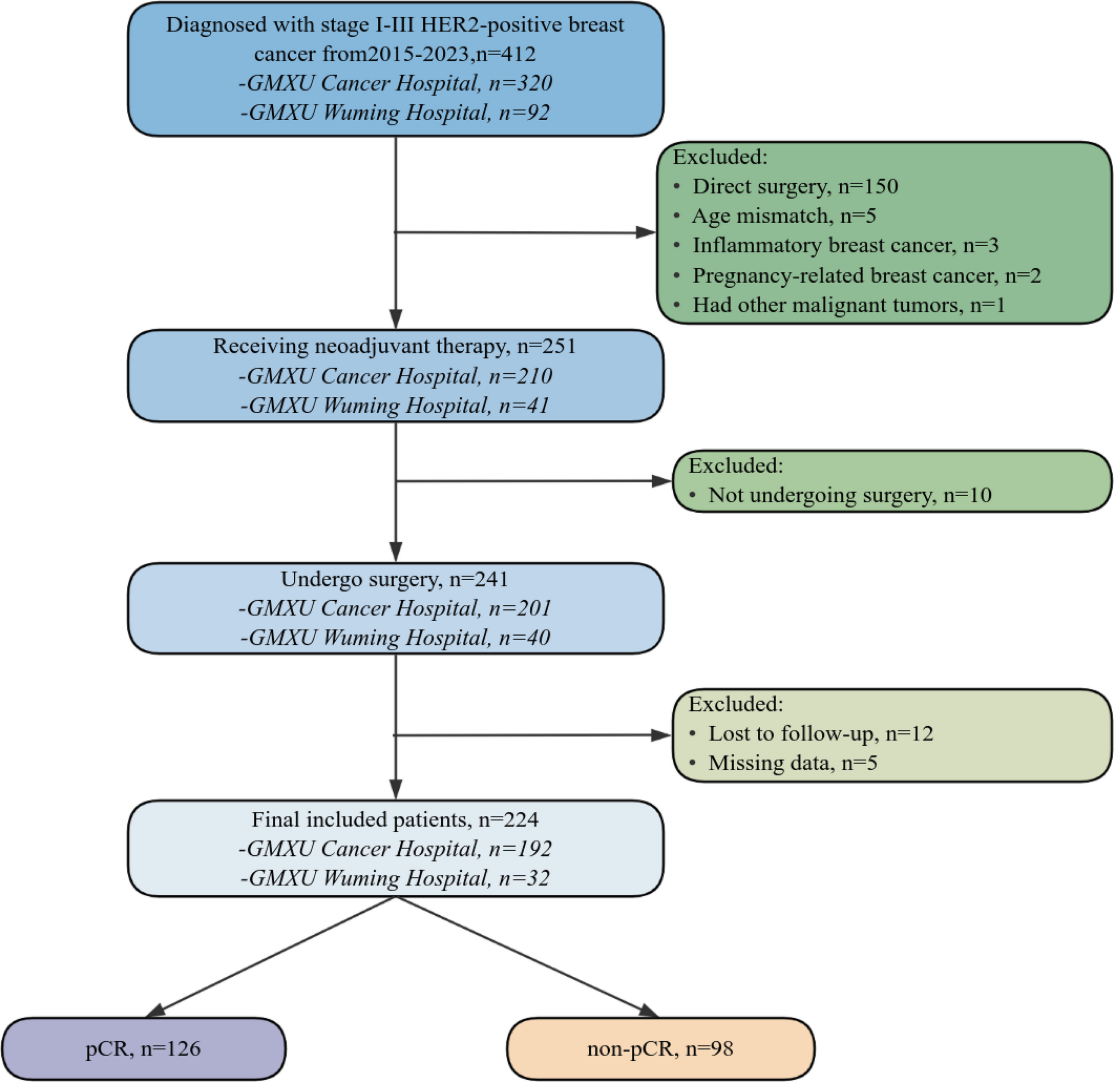
The case-screening flowchart. Numbers are presented in aggregate by institution to ensure data de-identification and patient confidentiality.

All procedures involving human participants were conducted in compliance with institutional guidelines, the ethical principles of the Declaration of Helsinki (1964) and subsequent amendments, and were approved by the respective institutional ethics committees. This retrospective, dual-center study included 224 patients with histologically confirmed HER2-positive invasive breast cancer who underwent NAC followed by surgery at Guangxi Medical University Cancer Hospital and Guangxi Medical University Wuming Hospital between January 1, 2015, and December 31, 2023. Patient demographics, clinicopathologic characteristics, laboratory tests, and treatment details were extracted from electronic medical records. In accordance with institutional ethics requirements, all patient data were fully de-identified prior to analysis, and institution-level identifiers were removed from the final analytic dataset. Case numbers by institution are therefore reported only in aggregate form in the case-screening flowchart. The case selection process is illustrated in **Figure 1**.

Inclusion criteria were: (1) Female patients aged 18–75 years; (2) Histologically confirmed HER2-positive invasive breast cancer by core needle biopsy; (3) Completion of NAC and definitive surgery at the participating institutions; (4) Availability of complete clinical and follow-up data.

Exclusion criteria were: (1) Prior chemotherapy, radiotherapy, endocrine therapy, or targeted therapy before NAC; (2) Patients with distant metastasis; (3) Patients with Inflammatory Breast Cancer; (4) History of other malignancies; (5) Receipt of blood transfusion within one month prior to NAC; (6) Patients with autoimmune diseases, chronic inflammation and/or cardiopulmonary diseases; (7) diagnosed with pregnancy-associated breast cancer.

### 2.2 Classification and assessment

Tumor staging was determined according to the 8th edition of the American Joint Committee on Cancer (AJCC) staging system (22). ER, PR, HER2, Ki-67, and EGFR status were assessed by immunohistochemistry (IHC) or fluorescence *in situ* hybridization (FISH) according to standardized protocols. ER/PR IHC staining with <1% positive neoplastic cells is defined as negative; IHC staining with 1-10% positive neoplastic cells is defined as low expression (23). HER-2 IHC scores of 0 or 1+ were considered negative; 3+ was positive; 2+ cases underwent FISH, with amplification confirming HER-2 positivity. Histological tumor response was graded using the Miller–Payne system (MPG). Pathological complete response (pCR) was defined as the absence of residual invasive carcinoma in both the breast and axillary lymph nodes (ypT0/Tis ypN0) (24).

Peripheral blood samples were collected within one week prior to initiating NAC. Complete blood counts were used to calculate the following systemic IIBs:


$$\text{NLR}\;=\;\frac{\text{Neutrophil count}}{\text{Lymphocyte count}}$$

$$\text{MLR}\;=\;\frac{\text{Monocyte count}}{\text{Lymphocyte count}}$$

$$\text{PLR}\;=\;\frac{\text{Platelet count}}{\text{Lymphocyte count}}$$

$$\text{SII}\;=\;\frac{\text{Platelet count}\times \text{Neutrophil count}}{\text{Lymphocyte count}}$$

### 2.3 Treatment regimens

Chemotherapy regimens were selected based on contemporaneous guideline recommendations and patient preferences. Preferred regimens incorporated HER2-targeted therapy (trastuzumab ± pertuzumab) combined with taxanes (T) and/or carboplatin (Cb), anthracyclines (A), and/or cyclophosphamide (C). Each cycle was repeated every three weeks, followed by a 2–3 week interval before surgery. For patients receiving targeted drug therapy, anti-HER2 therapy alone should be continued for a full year after surgery.

Chemotherapy regimens without HER2-targeted therapy include: AC-T regimen: anthracyclines 60mg/m2, cyclophosphamide 600mg/m2, followed by docetaxel 75mg/m2; TAC regimen: docetaxel 75mg/m2, anthracyclines 50mg/m2, and cyclophosphamide 600mg/m2.

Chemotherapy regimens using single-target therapy include: TH regimen: docetaxel 75mg/m2, trastuzumab 8mg/kg for the first time, then 6mg/kg.

Chemotherapy regimens using dual-target therapy include: THP regimen: paclitaxel liposomes 175mg/m2, trastuzumab 8mg/kg for the first time, then 6mg/kg and pertuzumab first dose 840mg, then 420mg; TCbHP regimen: paclitaxel liposomes 175mg/m2, carboplatin 400mg/m2, trastuzumab 8mg/kg for the first time, then 6mg/kg and pertuzumab first dose 840mg, then 420mg.

### 2.4 Follow-up and endpoints

Within 2 years after surgery, patients underwent outpatient follow-up assessments every 3 months, followed by semiannual assessments during the next 3–5 years, and annual comprehensive follow-up assessments beyond 5 years postoperatively until disease recurrence, death, or the study end date (April 30, 2025). Patients lost to follow-up were excluded from this study. Disease-free survival (DFS) was defined as the interval from the date of definitive surgery to the first documented local or regional recurrence, distant metastasis, or death from any cause. Distant metastasis–free survival (DMFS) was defined as the time from surgery to the occurrence of the first distant metastatic event. Overall survival (OS) was defined as the time from surgery to death from any cause.

### 2.5 Statistical analysis

Statistical analysis was performed using R 4.5.1 (R Project, Vienna, Austria) software. Variables with a missing value proportion greater than 20% were excluded, while variables with missing values less than or equal to 20% were processed using multiple imputation methods (**Supplementary Table 1**). Frequencies and percentages were used to describe categorical variables, with comparisons made using the chi-square test or Fisher’s exact test; median (interquartile range) or mean ± standard deviation were used to describe continuous variables, with comparisons made using the Mann-Whitney U test or t-test.

ROC curve analysis was used to evaluate predictive performance, with AUCs, sensitivities, and specificities reported together with 95% confidence intervals (CIs). For each immune–inflammatory biomarker (IIB), the optimal cut-off value was determined based on the maximal Youden index, and continuous variables were dichotomized into high and low groups. The stability of the ROC-derived cut-off values was assessed by estimating 95% confidence intervals using bootstrap resampling with 1,000 iterations. All ROC-based cut-off values were derived using pCR as the primary endpoint. These cut-offs were subsequently applied to DFS, DMFS, and OS analyses for consistency, rather than recalculated separately for time-to-event outcomes. The dichotomized IIB variables were subsequently entered into LASSO regression for feature selection and model construction. LASSO regression was performed using 10-fold cross-validation, in which the dataset was randomly partitioned into 10 subsets; in each iteration, nine subsets were used for model training and the remaining subset for validation, such that each subset served once as the validation set. The cross-validated mean binomial deviance was calculated across a sequence of penalty parameters (λ). Two commonly used criteria were considered: λ_min, defined as the λ yielding the minimum mean cross-validated deviance, and λ_1se, defined as the largest λ within one standard error of the minimum deviance. Variable selection was performed using the more conservative λ_1se criterion to obtain a parsimonious and interpretable model.

Survival outcomes were analyzed using the Kaplan–Meier method with comparisons conducted by the log-rank test. Variables achieving statistical significance in univariate Cox proportional hazards models (P<0.05) were further subjected to LASSO analysis to identify independent prognostic factors.

Subgroup analyses were stratified according to key clinicopathological covariates (age, tumor markers, clinical stage, hormone receptor status, Ki-67, NAC cycles and targeted Treatment regimen). pCR was analyzed using binary logistic regression; DFS was analyzed using the Cox proportional hazards model. Interaction terms and likelihood ratio tests were used to assess heterogeneity of effect across subgroups; P for interaction >0.05 was interpreted as evidence of consistent predictive value across strata.

## 3 Results

### 3.1 Clinicopathological characteristics

A total of 224 patients with HER2-positive breast cancer met eligibility criteria. At diagnosis, the mean patient age was 47.64 years. Body mass index (BMI) was categorized according to the Chinese adult BMI classification criteria, with BMI ≥24 kg/m² defined as overweight. High body mass index (BMI ≥ 24 kg/m²) was observed in 37.05% of patients, and 40.18% were premenopausal. The cut-off values for CA15-3 (25 ng/mL) and CEA (5 ng/mL) were defined based on the upper limits of normal routinely used in clinical practice and recommended by the assay manufacturers. Most patients had low baseline serum tumor marker levels, with CA15-3 ≤ 25 ng/mL in 85.71% and CEA ≤ 5 ng/mL in 84.38%. Clinically, the majority of cases presented with cT2 tumors (54.02%) and cN1 nodal involvement (54.91%). A total of 46.88% (n = 105) of patients were diagnosed with Stage III disease according to the cTNM classification. Low expression of ER was observed in 7.59% (n = 17) of cases, and low expression of PR in 6.70% (n = 15). Ki-67 was dichotomized using a cutoff value of 30%, which is commonly applied in clinical practice and previous studies to distinguish tumors with high proliferative activity (25). Patients with high proliferation (Ki-67 > 30%) accounted for 68.30%, those with Pathological grade II accounted for 58.48%, and EGFR-positive patients accounted for 57.14%. Approximately 69.64% of patients completed six or more cycles of treatment. Among these patients, 54.91% received dual HER2-targeted therapy(trastuzumab and pertuzumab), 29.46% were administered single-agent trastuzumab combined with a taxane, and 15.62% received chemotherapy alone without HER2-targeted therapy.

In the final pathological investigation after surgery, 126 cases (56.25%) of patients achieved pCR. Between pCR and non-pCR groups, significant differences were observed for CA15-3 (P = 0.004), cT stage (P = 0.022), cTNM stage (P = 0.004), total NAC cycles (P < 0.001), targeted therapy type (P < 0.001), NLR (P = 0.001), PLR (P < 0.001), and SII (P < 0.001) (**Table 1**).

**Table 1:** Clinicopathological characteristics of HER2-positive breast cancer patients.

Variables	Overall(n=224)	non-pCR(n=98)	pCR(n=126)	P-value
Age (mean (SD))	47.64 (9.80)	47.86 (9.68)	47.48 (9.92)	0.773
BMI(%)				0.613
<24	141 (62.95)	64 (65.31)	77 (61.11)	
≥24	83 (37.05)	34 (34.69)	49 (38.89)	
Menopausal status(%)				0.391
Premenopausal	134 (59.82)	55 (56.12)	79 (62.70)	
Postmenopausal	90 (40.18)	43 (43.88)	47 (37.30)	
CA15-3(%)				**0.004**
≤25	192 (85.71)	76 (77.55)	116 (92.06)	
>25	32 (14.29)	22 (22.45)	10 ( 7.94)	
CEA(%)				0.054
≤5	189 (84.38)	77 (78.57)	112 (88.89)	
>5	35 (15.62)	21 (21.43)	14 (11.11)	
cT stage(%)				**0.022**
1	20 ( 8.93)	8 ( 8.16)	12 ( 9.52)	
2	121 (54.02)	44 (44.90)	77 (61.11)	
3	50 (22.32)	31 (31.63)	19 (15.08)	
4	33 (14.73)	15 (15.31)	18 (14.29)	
cN stage(%)				0.058
0	47 (20.98)	14 (14.29)	33 (26.19)	
1	123 (54.91)	56 (57.14)	67 (53.17)	
2	42 (18.75)	24 (24.49)	18 (14.29)	
3	12 ( 5.36)	4 ( 4.08)	8 ( 6.35)	
cTNM stage(%)				**0.004**
I	3 ( 1.34)	3 ( 3.06)	0 ( 0.00)	
II	116 (51.79)	40 (40.82)	76 (60.32)	
III	105 (46.88)	55 (56.12)	50 (39.68)	
ER(%)				1
Negative	207 (92.41)	91 (92.86)	116 (92.06)	
Low expression	17 ( 7.59)	7 ( 7.14)	10 ( 7.94)	
PR(%)				0.567
Negative	209 (93.30)	93 (94.90)	116 (92.06)	
Low expression	15 ( 6.70)	5 ( 5.10)	10 ( 7.94)	
Ki-67(%)				0.899
≤30%	71 (31.70)	32 (32.65)	39 (30.95)	
>30%	153 (68.30)	66 (67.35)	87 (69.05)	
Pathological grading(%)				0.959
II	131 (58.48)	58 (59.18)	73 (57.94)	
III	93 (41.52)	40 (40.82)	53 (42.06)	
EGFR(%)				0.542
Negative	100 (44.64)	41 (41.84)	59 (46.83)	
Positive	124 (55.36)	57 (58.16)	67 (53.17)	
Cycle(%)				**<0.001**
<6	68 (30.36)	44 (44.90)	24 (19.05)	
≥6	156 (69.64)	54 (55.10)	102 (80.95)	
Targeted therapy(%)				**<0.001**
None	35 (15.62)	29 (29.59)	6 ( 4.76)	
Trastuzumab alone	66 (29.46)	35 (35.71)	31 (24.60)	
Trastuzumab and Pertuzumab	123 (54.91)	34 (34.69)	89 (70.63)	
NLR(%)				**0.001**
≤1.730	70 (31.25)	19 (19.39)	51 (40.48)	
>1.730	154 (68.75)	79 (80.61)	75 (59.52)	
PLR(%)				**<0.001**
≤200.315	167 (74.55)	55 (56.12)	112 (88.89)	
>200.315	57 (25.45)	43 (43.88)	14 (11.11)	
MLR(%)				0.133
≤0.195	103 (45.98)	39 (39.80)	64 (50.79)	
>0.195	121 (54.02)	59 (60.20)	62 (49.21)	
SII(%)				**<0.001**
≤723.380	143 (63.84)	42 (42.86)	101 (80.16)	
>723.380	81 (36.16)	56 (57.14)	25 (19.84)	

The bolded values indicate that the P-value < 0.05, indicating a statistically significant difference.

### 3.2 Correlation between IIBs and NAC response

**Figure 2:**
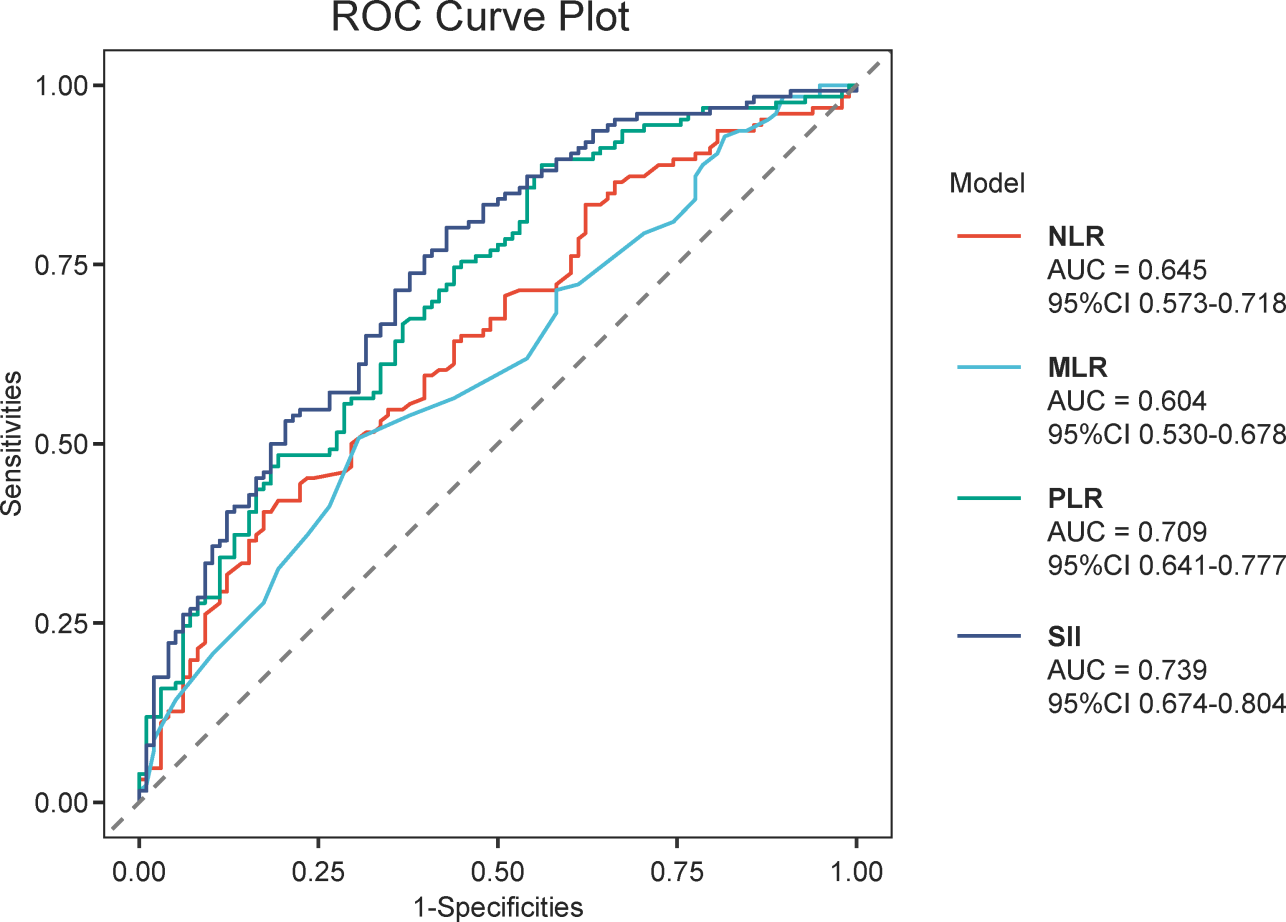
Predictive performance of IIBs for pCR in HER2-positive breast cancer after neoadjuvant chemotherapy. ROC curves were constructed to evaluate the discrimination ability of each IIB to predict pCR. The area under the ROC curve (AUC) quantifies overall predictive accuracy: higher AUC indicates better discrimination for predicting pCR. The optimal cutoff for each IIB was determined by the Youden index.

The optimal cut-off values of IIBs for predicting pCR were determined by ROC analysis, grouped by NLR (1.730), MLR (0.195), PLR (200.315), and SII (723.380). Corresponding AUC values were 0.645, 0.604, 0.709, and 0.739, respectively (**Table 2**, **Figure 2**). SII demonstrated the highest predictive performance among all IIBs.

**Table 2:** Receiver operating characteristic curve analyses for pathological complete response.

Curve	Cut-off value (95%CI)	AUC (95%CI)	Sensitivity (95%CI)	Specificity (95%CI)	P-value
NLR	1.730 (1.580–3.040)	0.645 (0.573-0.718)	0.405 (0.325-0.905)	0.827 (0.326-0.908)	0.005
MLR	0.195 (0.145–0.345)	0.604 (0.530-0.678)	0.508 (0.262-0.952)	0.694 (0.194-0.898)	0.003
PLR	200.315 (132.420–205.255)	0.709 (0.641-0.777)	0.889 (0.452-0.937)	0.439 (0.388-0.878)	<0.001
SII	723.380 (499.560–894.360)	0.739 (0.674-0.804)	0.802 (0.500-0.937)	0.571 (0.429-0.867)	<0.001

**Figure 3:**
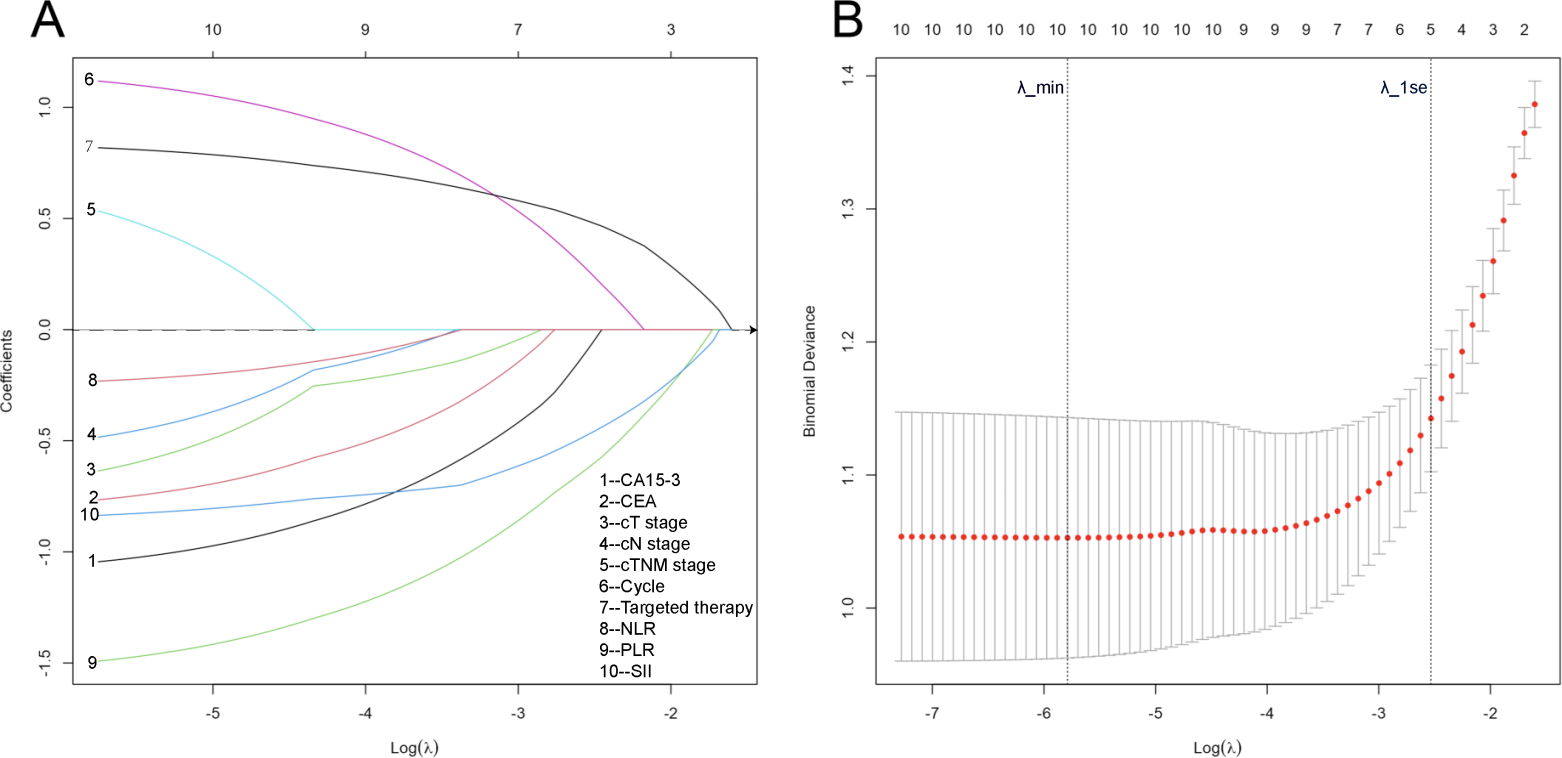
Results of LASSO regression and 10-fold cross-validation for the outcome of pCR. **(A)** LASSO coefficient paths of candidate predictors plotted against log(λ). The y-axis shows coefficient estimates; increasing λ shrinks coefficients toward zero (horizontal line at coefficient = 0), and predictors with coefficients reduced to zero are excluded. Numbers on the top x-axis indicate the number of predictors with non-zero coefficients at each λ. **(B)** 10-fold cross-validation curve of the LASSO model showing the mean cross-validated binomial deviance ( ± 1 standard error) across a sequence of λ values. Each point represents the mean deviance across the 10 folds at a given λ. Vertical dotted lines indicate λ_min (the value of λ corresponding to the minimum mean cross-validated deviance) and λ_1se (the largest λ within one standard error of the minimum deviance). Variable selection was performed using the more conservative λ_1se criterion (λ_1se = 0.0795), under which five predictors retained non-zero coefficients. Numbers above the plot indicate the number of predictors with non-zero coefficients at each λ.

In univariate logistic regression, low NLR (OR = 0.35, 95% CI 0.19-0.65, P < 0.001), low PLR (OR = 0.16, 95% CI 0.08-0.32, P < 0.001), and low SII (OR = 0.19, 95% CI 0.10-0.34, P < 0.001) predicted a higher likelihood of achieving pCR. Additional predictors included low CA15-3 (OR = 0.30, 95% CI 0.13-0.66, P = 0.003), low CEA (OR = 0.46, 95% CI 0.22-0.96, P = 0.038), earlier clinical stage (OR = 0.51, 95% CI 0.30-0.88, P = 0.015), ≥6 NAC cycles (OR = 0.47, 95% CI 0.24-0.94, P < 0.001), single-target (OR = 4.28, 95% CI 1.57-11.67, P = 0.004) or dual-target (OR = 12.65, 95% CI 4.83-33.17, P < 0.001) anti HER2 therapy(**Table 3**). The 10 variables that were statistically significant in the univariate logistic analysis were included in the LASSO regression analysis, and the optimal λ value was selected through 10-fold cross-validation (**Figure 3**). Although λ_min (0.0031) indicated the model with the minimum cross-validated deviance, variable selection was performed using the more conservative λ_1se criterion (λ_1se = 0.0795) to obtain a parsimonious and interpretable model. Under λ_1se, five variables retained non-zero coefficients and were therefore identified as key predictors of pCR: SII, PLR, CA15-3, number of NAC cycles, and targeted therapy (**Supplementary Table 2**).

**Table 3:** Univariate logistic regression of clinical characteristics and IIBs in relation to pCR.

Variables	Univariate analysis OR(95% CI)	P-value
Age	1.00 (0.97-1.02)	0.772
BMI		
<24	Ref.	
≥24	1.20 (0.69-2.07)	0.519
Menopausal status		
Premenopausal	Ref.	
Postmenopausal	0.76 (0.44-1.30)	0.32
CA15-3		
≤25	Ref.	
>25	0.30 (0.13-0.66)	**0.003**
CEA		
≤5	Ref.	
>5	0.46 (0.22-0.96)	**0.038**
cT stage		
T1/2	Ref.	
T3/4	0.47 (0.27-0.82)	**0.007**
cN stage		
N0	Ref.	
N+	0.47 (0.24-0.94)	**0.032**
cTNM stage		
I-II	Ref.	
III	0.51 (0.30-0.88)	**0.015**
ER		
Negative	Ref.	
Low expression	1.12 (0.41-3.06)	0.824
PR		
Negative	Ref.	
Low expression	1.60 (0.53-4.85)	0.403
Ki-67		
≤30%	Ref.	
>30%	1.08 (0.61-1.91)	0.786
Pathological grading		
II	Ref.	
III	0.87 (0.51-1.48)	0.609
EGFR		
Negative	Ref.	
Positive	0.66 (0.39-1.13)	0.129
Cycle		
<6	Ref.	
≥6	3.46 (1.91-6.29)	**<0.001**
Targeted therapy		
None	Ref.	
Trastuzumab alone	4.28 (1.57-11.67)	**0.004**
Trastuzumab and Pertuzumab	12.65 (4.83-33.17)	**<0.001**
NLR		
≤1.730	Ref.	
>1.730	0.35 (0.19-0.65)	**<0.001**
PLR		
≤200.315	Ref.	
>200.315	0.16 (0.08-0.32)	**<0.001**
MLR		
≤0.195	Ref.	
>0.195	0.64 (0.38-1.09)	0.102
SII		
≤723.380	Ref.	
>723.380	0.19 (0.10-0.34)	**<0.001**

The bolded values indicate that the P-value < 0.05, indicating a statistically significant difference.

Based on univariate logistic regression and LASSO regression, SII, PLR, CA15-3, CEA, number of neoadjuvant therapies and targeted therapy status were predictors of pCR. Notably, when integrating ROC performance, SII (AUC = 0.739) outperformed NLR (AUC = 0.709) in predicting pCR (**Figure 2**).

### 3.3 Correlation between IIBs and DFS

**Figure 4:**
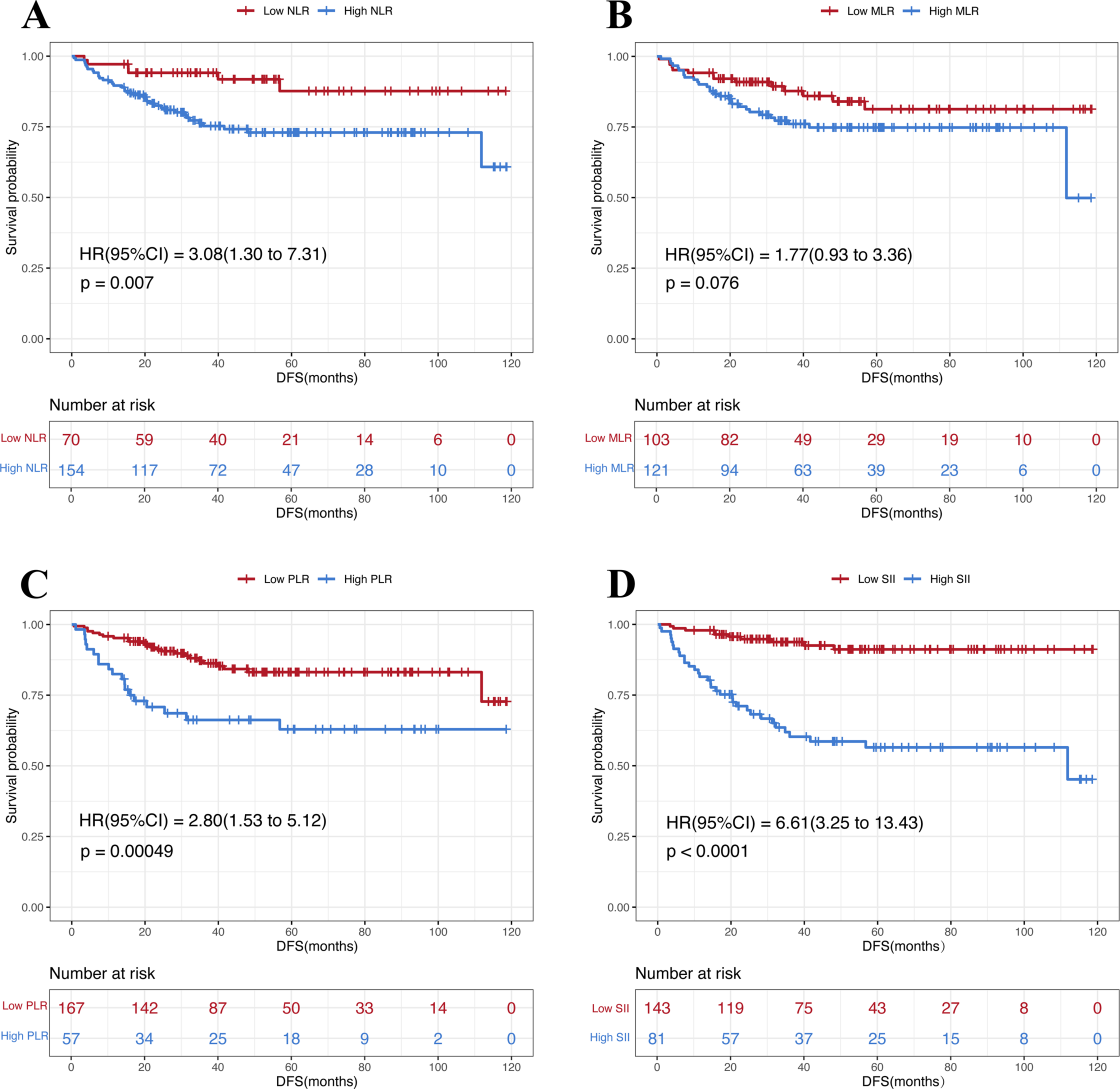
Kaplan-Meier analysis of the relationship between IIBs and DFS. **(A)** NLR; **(B)** MLR; **(C)** PLR; **(D)** SII.

The mean follow-up duration was 47.1 months. During follow-up, a total of 43 DFS events were observed. Kaplan–Meier analyses revealed that patients with low NLR, low PLR and low SII before NAC had significantly longer DFS compared to those with higher values (NLR: P = 0.0007; PLR: P = 0.00049; SII: P < 0.0001). MLR showed no statistically significant association with DFS (P = 0.076) (**Figure 4**).

**Figure 5:**
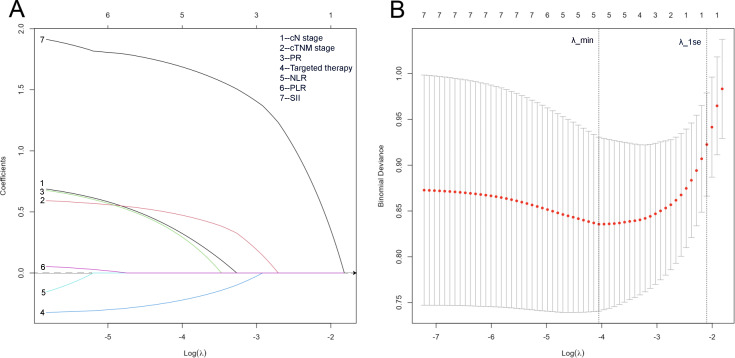
Results of LASSO regression and 10-fold cross-validation for the outcome of DFS. **(A)** LASSO coefficient paths plotted against log(λ). Coefficients are progressively shrunk toward zero as λ increases (horizontal line at coefficient = 0), and predictors with coefficients reduced to zero are excluded. Numbers on the top x-axis indicate the number of predictors with non-zero coefficients at each λ. **(B)** Ten-fold cross-validation curve showing the mean cross-validated binomial deviance ( ± 1 SE) across a sequence of λ values. Vertical dotted lines indicate λ_min and λ_1se; the optimal λ was selected as λ_min = 0.0174. While λ_min (0.0174) indicates the model with optimal predictive performance, variable selection was performed using the more conservative λ_1se criterion (λ_1se = 0.1227), under which only SII retained a non-zero coefficient.

In univariate Cox regression, positive nodal status (N+), cTNM stage III, PR low expression, high NLR, high PLR, and high SII were associated with increased recurrence/metastasis risk, whereas dual-target anti-HER2 therapy significantly reduced risk (**Table 4**). The 7 variables that were statistically significant in the univariate Cox regression analysis were entered into the LASSO Cox regression model, and the penalty parameter (λ) was determined using 10-fold cross-validation (**Figure 5**). Under λ_1se = 0.1227, only SII retained a non-zero coefficient and was therefore identified as the sole variable independently associated with DFS (**Supplementary Table 3**). Patients in the low SII group exhibited significantly prolonged DFS.

**Table 4:** Univariate COX analysis of clinicopathological characteristics and IIBs in relation to DFS.

Variables	Univariate analysis HR(95%CI)	P-value
Age	0.996 (0.966, 1.027)	0.81
BMI		
<24	Ref.	
≥24	1.368 (0.749, 2.497)	0.308
Menopausal status		
Premenopausal	Ref.	
Postmenopausal	1.096 (0.598, 2.009)	0.766
CA15-3		
≤25	Ref.	
>25	1.177 (0.523, 2.646)	0.694
CEA		
≤5	Ref.	
>5	0.803 (0.339, 1.905)	0.619
cT stage		
T1/2	Ref.	
T3/4	1.598 (0.878, 2.909)	0.125
cN stage		
N0	Ref.	
N+	3.863 (1.195, 12.489)	**0.024**
cTNM stage		
I-II	Ref.	
III	2.670 (1.391, 5.124)	**0.003**
ER		
Negative	Ref.	
Low expression	1.517 (0.538, 4.278)	0.431
PR		
Negative	Ref.	
Low expression	2.579 (1.009, 6.594)	**0.048**
Ki-67		
≤30%	Ref.	
>30%	1.278 (0.656, 2.491)	0.47
Pathological grading		
II	Ref.	
III	1.568 (0.861, 2.853)	0.141
EGFR		
Negative	Ref.	
Positive	0.964 (0.530, 1.756)	0.906
Cycle		
<6	Ref.	
≥6	0.654 (0.355, 1.205)	0.174
Targeted therapy		
None	Ref.	
Trastuzumab alone	0.852 (0.405, 1.792)	0.674
Trastuzumab and Pertuzumab	0.379 (0.168, 0.853)	**0.019**
NLR		
≤1.730	Ref.	
>1.730	3.084 (1.301, 7.310)	**0.011**
PLR		
≤200.315	Ref.	
>200.315	2.798 (1.529, 5.117)	**0.001**
MLR		
≤0.195	Ref.	
>0.195	1.772 (0.935, 3.358)	**0.08**
SII		
≤723.380	Ref.	
>723.380	6.611 (3.255, 13.429)	**<0.001**

The bolded values indicate that the P-value < 0.05, indicating a statistically significant difference.

In addition, we explored DMFS and OS as secondary outcomes. Kaplan–Meier analyses showed a consistent trend toward poorer DMFS and OS in patients with high baseline SII (**Supplementary Figures S1**, **S2**). During follow-up, a total of 37 DMFS events and 13 OS events were recorded. However, due to the relatively limited number of DMFS and OS events, the confidence intervals were wide and the survival data remain immature, and these findings should therefore be interpreted with caution.

### 3.4 Subgroup analyses

To assess the robustness of SII as a predictor, subgroup analyses were performed stratified by age (<40 vs ≥40 years), CA15-3 (≤25ng/mL vs >25ng/mL), CEA (≤5ng/L vs >5ng/L), cT stage (T1/2 vs T3/4), cN stage (N0 vs N+), cTNM stage (I-II vs III), ER/PR status (Negative vs Low expression), Ki-67 (≤30% vs >30%), NAC cycles (<6 vs ≥6) and targeted therapy type (None, Trastuzumab alone, Trastuzumab and Pertuzumab).

**Figure 6:**
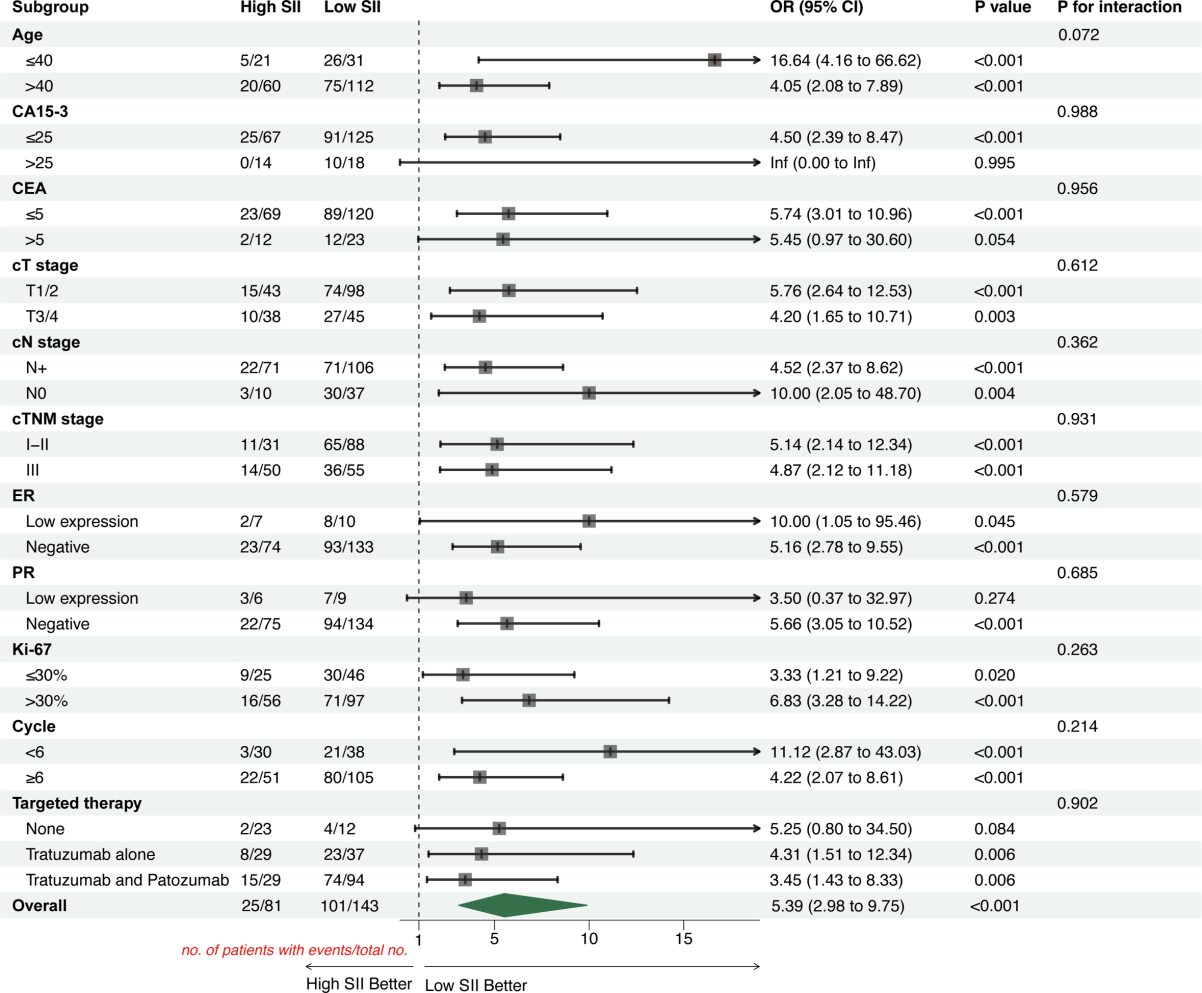
Forest plot for subgroup analysis of clinicopathological characteristics and treatment modalities of HER2-positive breast cancer patients (outcome: pCR). ORs with 95%CIs for achieving pCR are shown for high vs low SII across predefined subgroups. Points represent ORs and horizontal lines indicate 95% CIs; the vertical dashed line at OR = 1.0 denotes no association. The interaction p-value was calculated by adding an interaction term between SII group and each subgroup variable in the logistic regression model and tests whether the association between SII and pCR differs across subgroup levels (P for interaction > 0.05 indicates no evidence of effect modification across subgroups). The “Overall” estimate represents the effect of SII in the entire cohort.

**Figure 7:**
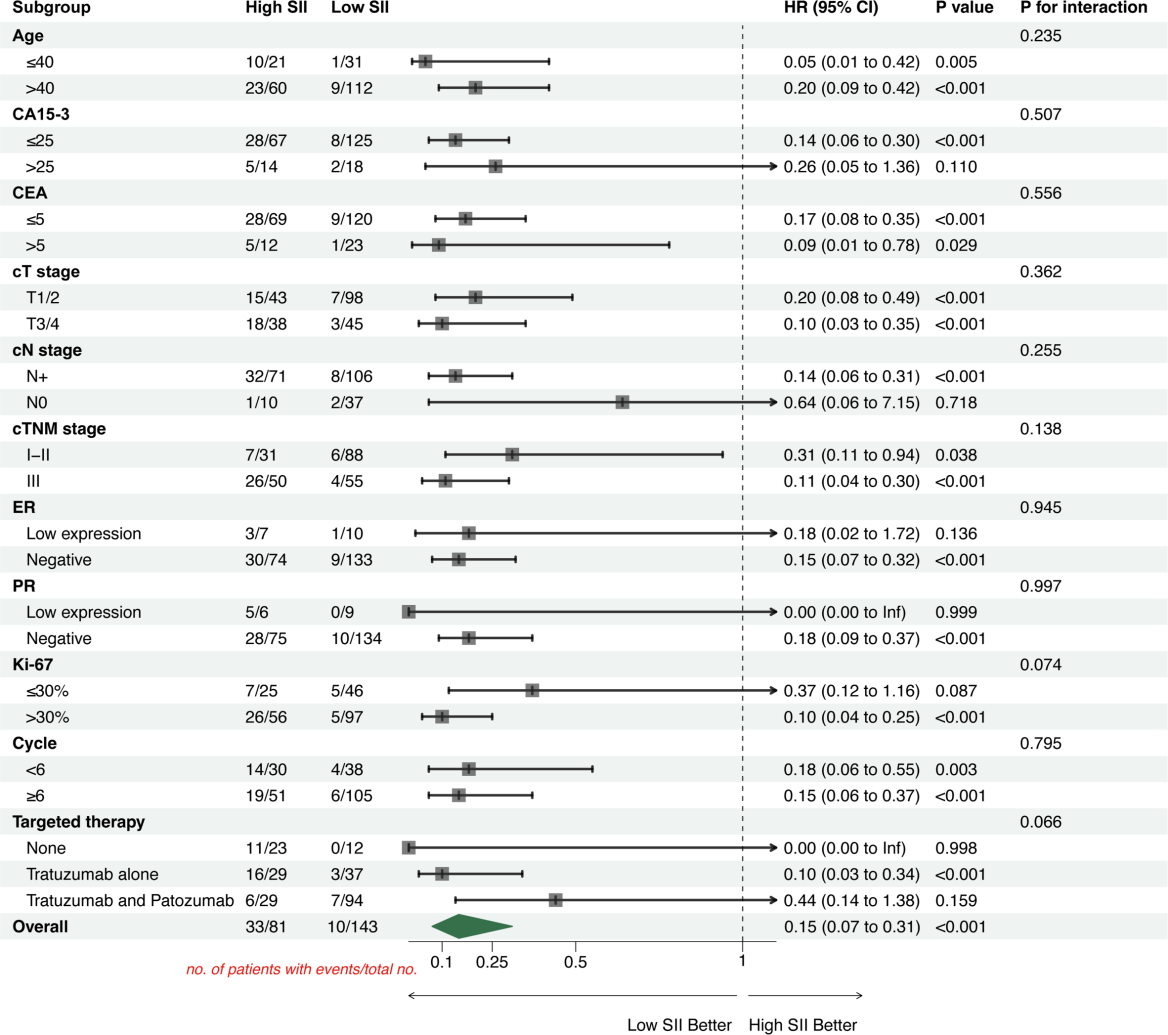
Forest plot for subgroup analysis of clinicopathological characteristics and treatment modalities of HER2-positive breast cancer patients (outcome: DFS). HRs with 95%CIs for DFS are presented for high vs low SII within each subgroup. Points represent HRs and horizontal lines indicate 95% CIs; the vertical dashed line at HR = 1.0 denotes no difference in hazard. The interaction p-value was obtained by including an interaction term between SII group and each subgroup variable in the Cox proportional hazards model and tests whether the prognostic effect of SII on DFS differs across subgroup levels (P for interaction > 0.05 indicates no evidence of effect modification across subgroups). The “Overall” estimate represents the effect of SII in the entire cohort.

Subgroup analysis results showed that patients with low SII were more likely to achieve pCR (**Figure 6**: Overall OR = 5.39, 95% CI 2.98–9.75, P<0.001), while patients with high SII had a significantly increased risk of relapse or progression compared to those with low SII (**Figure 7**: Overall HR = 0.15, 95% CI 0.07–0.31, P<0.001; HR<1 indicates better prognosis for low SII). The interaction test showed that there was no statistically significant interaction of SII with pCR and DFS among the subgroups (all P for interaction >0.05).

## 4 Discussion

This study systematically evaluated the predictive value of four readily obtainable peripheral IIBs for pCR and DFS in HER2-positive breast cancer patients receiving NAC. For the first time in this specific patient population, we confirmed that the Systemic immune-inflammation index (SII), which integrates neutrophils, thrombocytes, and lymphocyte count, is a more effective and robust independent indicator for predicting pCR and DFS compared to traditional markers such as NLR, PLR, and MLR.

Our findings underscore the intricate interplay between the tumor microenvironment (TME) and the host’s systemic immune–inflammatory status, and its critical influence on tumor response and long-term outcomes (11). In recent years, researchers have gradually recognized that systemic hematological indicators reflecting systemic inflammation and immune status can serve as intuitive, convenient, and reproducible prognostic tools. As a comprehensive indicator, the superior predictive ability of SII stems from its more comprehensive reflection of the imbalance between anti-tumor immunity and pro-tumor inflammation, thereby demonstrating better predictive capability in prognostic assessment (15). At the cellular mechanism level, neutrophils can directly promote tumor proliferation and metastasis by secreting growth factors, angiogenesis factors, and neutrophil extracellular traps (NETs); platelets assist circulating tumor cells in evading surveillance by the immune system and promote their colonization in distant organs by forming a protective “biological barrier,” thereby creating conditions for the formation of metastatic lesions; whereas lymphocytes are the core executors of anti-tumor immunity, playing a key role in tumor immune surveillance and clearance—they directly kill tumor cells through cytotoxic responses and the secretion of various cytokines, maintaining the body’s anti-tumor immune barrier (26–28). An elevated SII thus reflects a hyper-inflammatory, immunosuppressive systemic milieu—a state likely to blunt chemotherapy- and HER2-targeted therapy–driven anti-tumor immune responses, reduce pCR rates, and create a fertile “soil” for postoperative recurrence (29, 30). In this study, SII emerged as an independent predictor of pCR and DFS in both univariate logistic/Cox regression combined with LASSO regression analysis, which also strongly supports the aforementioned hypothesis from a mechanistic logic perspective. In other words, SII is not merely a hematological indicator but also a “window” reflecting the balance between the tumor microenvironment and host immune-inflammatory response, holding significant potential value in clinical prognosis assessment and personalized treatment decision-making.

The findings of this study are consistent with trends in previous literature but also advance key aspects. Numerous prior studies have confirmed the predictive and prognostic value of NLR in various solid tumors (including breast carcinoma) (31, 32). However, these studies often show inconsistent conclusions regarding HER-2-positive breast carcinoma, particularly in the context of neoadjuvant therapy, partly due to sample heterogeneity, differing endpoint definitions, and the failure to systematically compare multiple emerging indicators. In recent years, numerous academic works have discussed the prognostic value of SII in various cancers. The study by Passardi A indicated that patients with high SII had lower Progression-Free Survival and OS, suggesting a poor prognosis, which is particularly applicable in patients with advanced-stage colorectal carcinoma treated with bevacizumab combined with first-line chemotherapy (20). The research by Meng L demonstrated that high SII was associated with poorer OS, and SII might serve as an important prognostic indicator for patients with prostatic carcinoma (33). The study by Li D revealed that a higher SII was correlated with worse survival in Hepatocellular carcinoma patients after hepatic artery embolism arterial (21). Through a propensity score matching study, Xin Hua found that in the operable breast carcinoma population, preoperative SII could be a reliable predictor for OS and Distant Metastasis-Free Survival (34). The meta-analysis by Zhang Y revealed that elevated SII predicts poorer survival outcomes and is associated with Clinicopathological features indicative of Breast carcinoma progression (35). However, no study to date had rigorously examined its predictive performance for pCR and DFS in HER2-positive NAC-treated cohorts.

Our study makes a substantive contribution by evaluating a molecularly homogeneous and clinically well-defined HER2-positive population, employing a rigorous analytic framework that integrates univariate logistic and Cox regression with LASSO penalization to minimize overfitting, and confirming the robustness of SII’s predictive value across ten clinically relevant subgroups—thereby reinforcing the external validity of our findings.

The present findings carry notable clinical relevance. As a biomarker derived from routine complete blood counts, the SII offers a cost-effective, non-invasive, and universally accessible approach for baseline risk stratification in HER2-positive breast cancer prior to NAC (36). Patients with high pre-treatment SII may be considered for treatment intensification, such as escalation of chemotherapy regimens, incorporation of novel targeted combinations, or enrollment in clinical trials aimed at overcoming therapeutic resistance (37, 38). Conversely, patients with low SII probability of pCR could potentially be spared unnecessary chemotherapy toxicity through carefully considered de-escalation strategies without compromising efficacy. Furthermore, whether monitoring the dynamic changes of SII during treatment can more accurately predict treatment response will be an extremely attractive direction for future research.

In addition, this study also showed that pre-treatment CA15–3 and CEA levels, number of neoadjuvant therapies, targeted treatment strategy, and Surgical approach were all independent predictors of pCR (**Figure 4**). Lower levels of CA15–3 and CEA often reflect lower tumor burden and more indolent disease biology, which is consistent with the findings of Rafael Molina (39). Undergoing ≥6 cycles of neoadjuvant therapy may provide more extensive drug exposure and enhanced tumoricidal effects, particularly within the context of combination targeted therapy, which can significantly increase the pCR rate (40). Among these regimens, the dual-target therapy has demonstrated advantages in suppressing the HER2-signaling pathway and overcoming drug resistance, as confirmed in the NeoSphere study (41). Interestingly, breast-conserving surgery was more common among patients attaining pCR. This may reflect that these patients already had more favorable tumor staging and biological traits prior to treatment, rather than the Surgical approach itself improving the response rate (42). Although it was an independent predictor of pCR in the analysis, potential selection bias requires caution, and the mechanisms behind it warrant further investigation.

Although this study yielded important findings, several limitations should be acknowledged. First, as a retrospective dual-center study, it is inevitably subject to selection and information bias, and potential regional and institutional differences may limit the generalizability of our conclusions. Second, during the 10-year accrual period, the accessibility of HER2-targeted agents and neoadjuvant chemotherapy regimens in China underwent substantial changes, leading to treatment heterogeneity and introducing potential socioeconomic confounding. Despite statistical adjustments, these effects could not be completely eliminated. To mitigate such bias, we performed subgroup analyses stratified by treatment type, which showed that the prognostic impact of SII persisted across different regimens, although the strength of association varied. However, the sample size of patients without targeted therapy was limited, which produced a wider confidence interval in subgroup analyses. Therefore, the lack of statistically significant interactions should not be interpreted as equivalent proof, and the heterogeneity of residual effects cannot be ruled out. Third, residual confounding caused by unmeasured or incompletely measured factors—such as comorbidity burden and psychosocial stress or depression—remains possible. Fourth, SII was assessed only once prior to NAC, without evaluation of dynamic changes, and the ROC-derived cut-off requires external validation. In addition, although DMFS and OS were explored as secondary outcomes, the number of distant metastasis and death events was limited during the current follow-up period; therefore, these results remain immature and should be interpreted with caution. In summary, this study warrants confirmation in prospective, homogeneous, multicenter investigations with standardized regimens and broader population representation.In this dual-center retrospective cohort, the systemic immune-inflammation index (SII) showed the strongest association among tested inflammatory indices with both pathological complete response and disease-free survival in HER2-positive breast cancer receiving neoadjuvant therapy. As a low-cost metric from routine blood counts, SII may aid pre-treatment risk stratification. Prospective, homogeneous studies with standardized regimens, external validation, and longitudinal SII assessment are needed before clinical adoption.

## 5 Conclusion

In this dual-center retrospective cohort, the systemic immune-inflammation index (SII) showed the strongest association among tested inflammatory indices with both pathological complete response and disease-free survival in HER2-positive breast cancer receiving neoadjuvant therapy. As a low-cost metric from routine blood counts, SII may aid pre-treatment risk stratification. Prospective, homogeneous studies with standardized regimens, external validation, and longitudinal SII assessment are needed before clinical adoption.

## Data Availability

The raw data supporting the conclusions of this article will be made available by the authors, without undue reservation.

## Supplementary material

Supplementary Figure 1 Kaplan-Meier analysis of the relationship between IIBs and DMFS. **(A)** NLR; **(B)** MLR; **(C)** PLR; **(D)** SII.

Supplementary Figure 2 Kaplan-Meier analysis of the relationship between IIBs and OS. **(A)** NLR; **(B)** MLR; **(C)** PLR; **(D)** SII.
