# Supplementary figures and images for: Association of immuno-inflammatory biomarkers with response to neoadjuvant chemotherapy and prognosis in HER2-positive breast cancer: dual-center clinical evidence
Source: Front Immunol. 2026 Feb 9;17:1751072. doi: 10.3389/fimmu.2026.1751072 (PMC12926385; doi:10.3389/fimmu.2026.1751072)

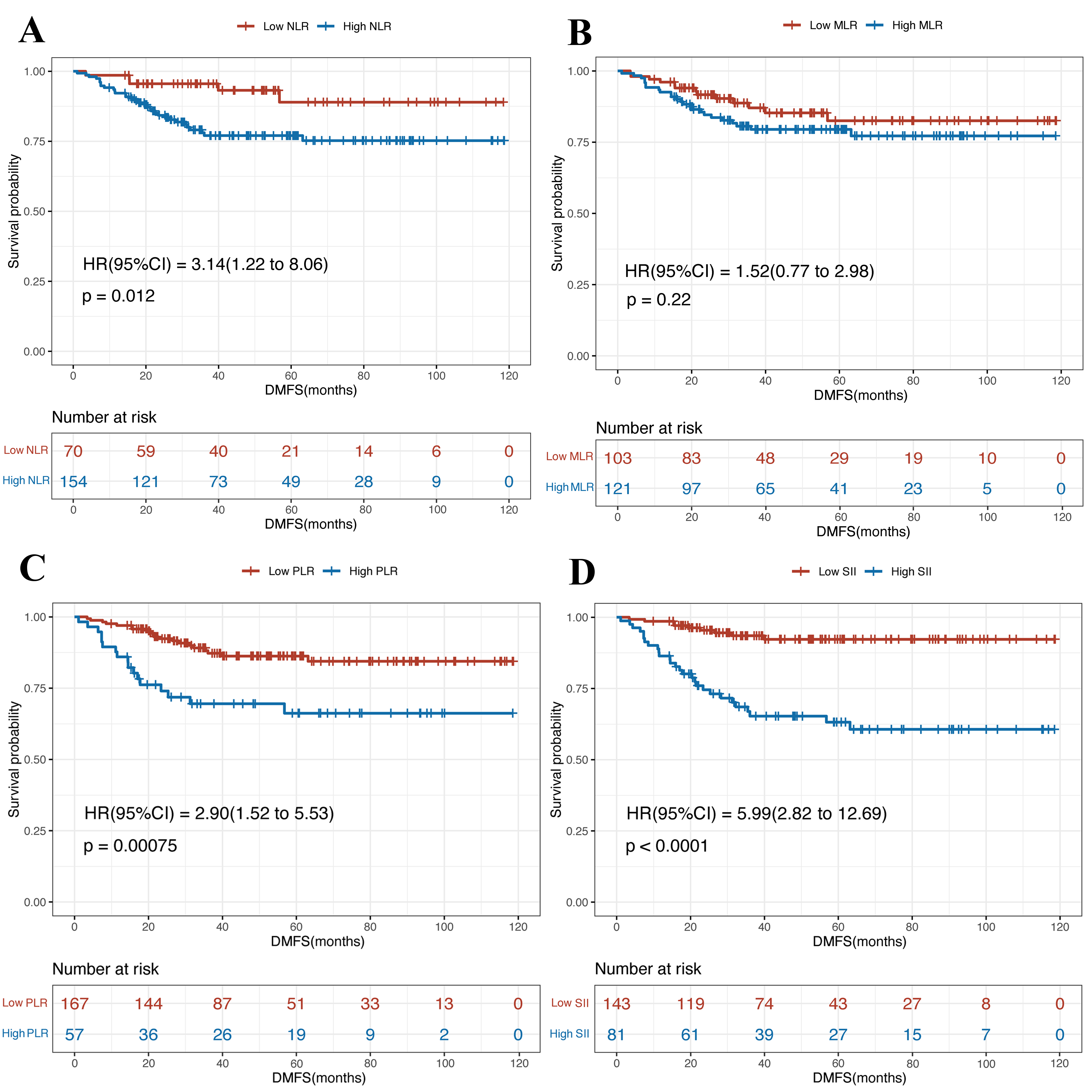

Supplement: Supplementary Figure 1 — Kaplan-Meier analysis of the relationship between IIBs and DMFS. (A) NLR; (B) MLR; (C) PLR; (D) SII. [file Image1.tif]

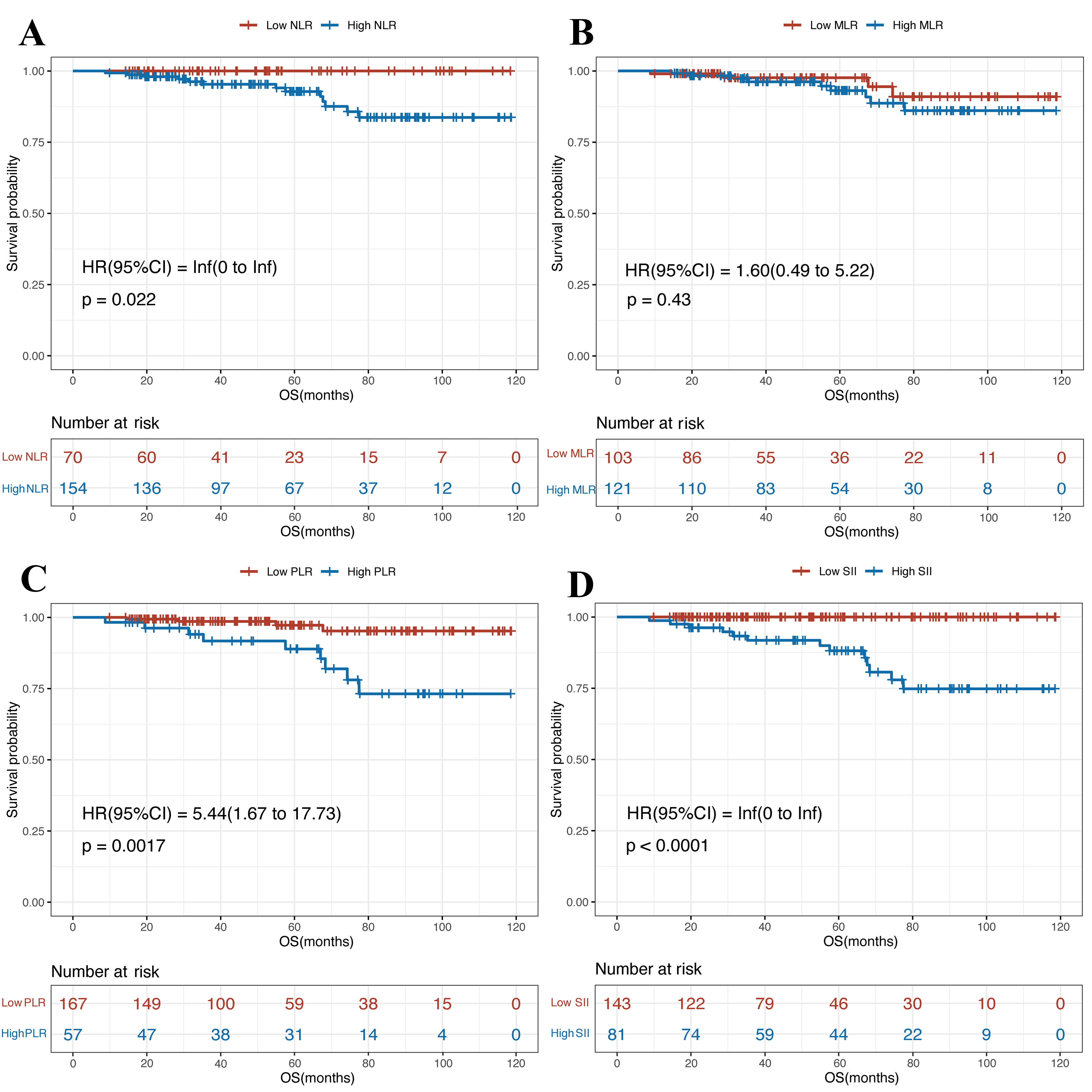

Supplement: Supplementary Figure 2 — Kaplan-Meier analysis of the relationship between IIBs and OS. (A) NLR; (B) MLR; (C) PLR; (D) SII. [file Image2.tif]
