# Supplementary material for: Association of immuno-inflammatory biomarkers with response to neoadjuvant chemotherapy and prognosis in HER2-positive breast cancer: dual-center clinical evidence
Source: Front Immunol. 2026 Feb 9;17:1751072. doi: 10.3389/fimmu.2026.1751072 (PMC12926385; doi:10.3389/fimmu.2026.1751072)
Supplement: Supplementary file 3 [file Table1.docx]

**Supplementary Table 1** Ratio of missing values of each variable.

| **Variable** | **Missing Ratio** |
| --- | --- |
| Age | 0 |
| BMI | 0 |
| Menopausal status | 0 |
| Ca-153 | 0 |
| CEA | 0 |
| cT stage | 0 |
| cN stage | 0 |
| cTNM stage | 0 |
| ER | 0 |
| PR | 0 |
| Ki-67 | 0 |
| Pathological grading | 0.143 |
| EGFR | 0.183 |
| Cycle | 0 |
| Targeted therapy | 0 |
| NLR | 0 |
| PLR | 0 |
| MLR | 0 |
| SII | 0 |

**Supplementary Table 2** LASSO regression screening for variables. (outcome :pCR)

| **Variables** | **lambda.1se** |
| --- | --- |
| (Intercept) | 0.161 |
| CA15-3 | -0.078 |
| CEA | 0 |
| cT stage | 0 |
| cN stage | 0 |
| cTNM stage | 0 |
| Cycle | 0.259 |
| Targeted therapy | 0.485 |
| NLR | 0 |
| PLR | -0.616 |
| SII | -0.468 |

**Supplementary Table 3** LASSO regression screening for variables. (outcome :DFS)

| **Variables** | **lambda.1se** |
| --- | --- |
| (Intercept) | -2.155 |
| cN stage | 0 |
| cTNM stage | 0 |
| PR | 0 |
| Targeted therapy | 0 |
| NLR | 0 |
| PLR | 0 |
| SII | 0.513 |
